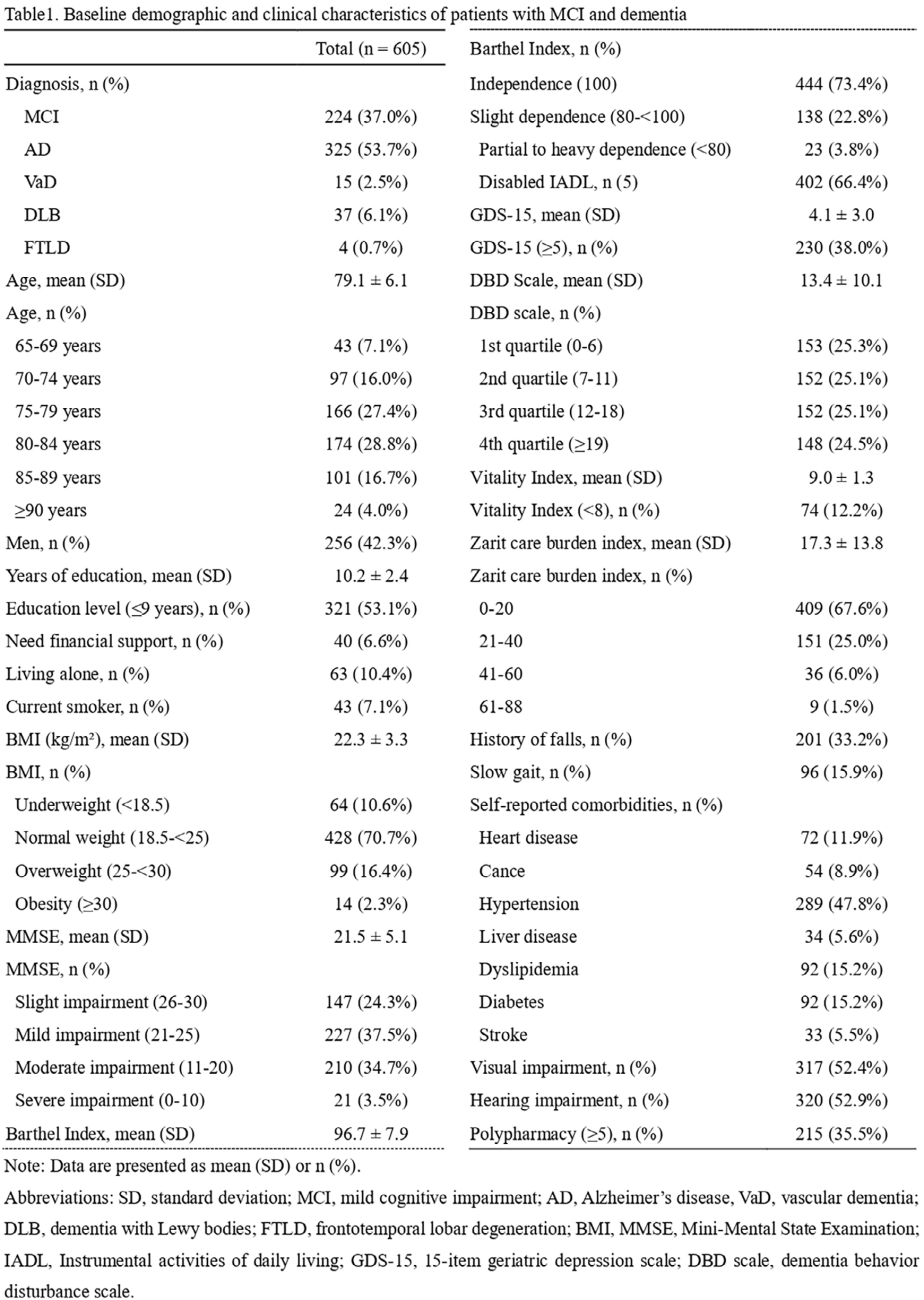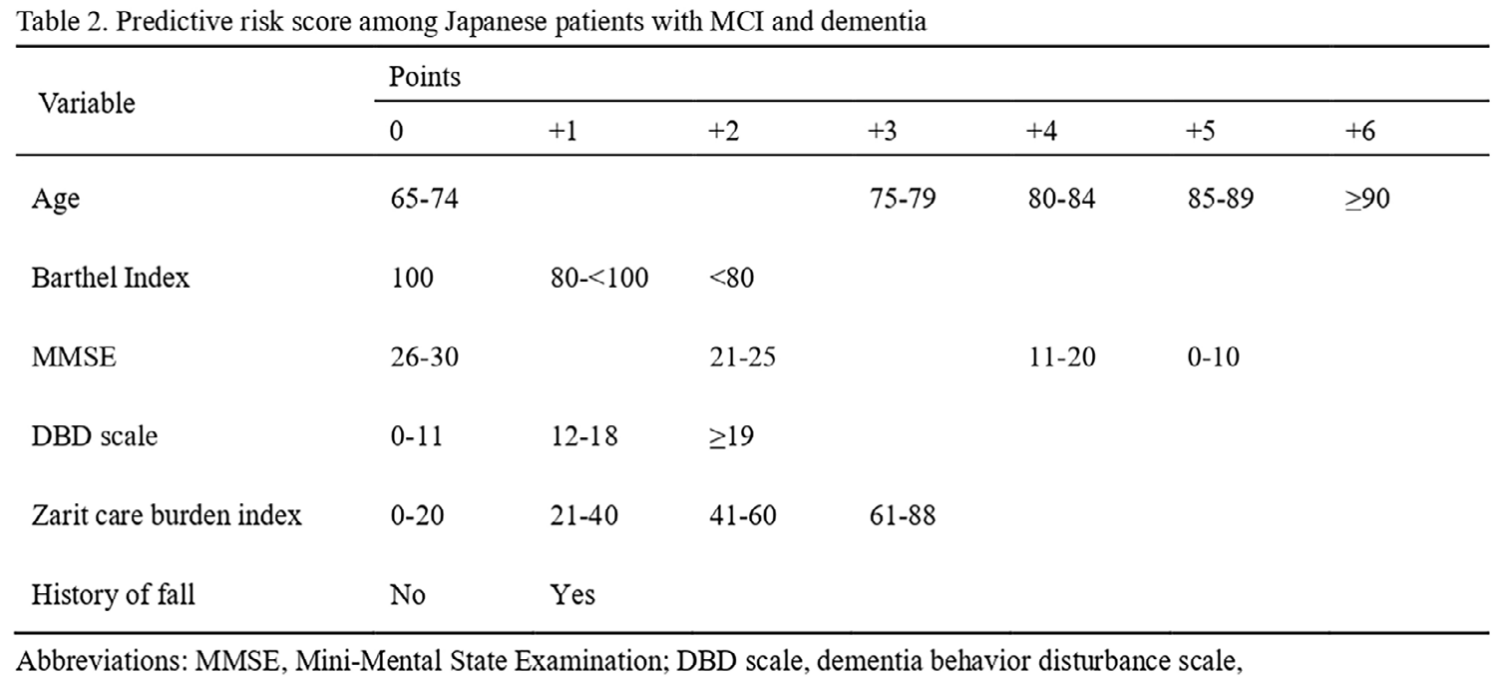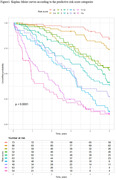# Developing a predictive model for deterioration in long‐term care level among Japanese older adults with mild cognitive impairment and dementia: NCGG‐STORIES

**DOI:** 10.1002/alz.088462

**Published:** 2025-01-09

**Authors:** Kazuaki Uchida, Taiki Sugimoto, Xueying Jin, Takeshi Nakagawa, Soshiro Ogata, Eri Kiyoshige, Kosuke Fujita, Yujiro Kuroda, Takashi Sakurai, Tami Saito

**Affiliations:** ^1^ National Center for Geriatrics and Gerontology, Obu, Aichi Japan; ^2^ University of Washington, Seattle, WA USA; ^3^ National Cerebral and Cardiovascular Center, Suita, Osaka Japan

## Abstract

**Background:**

To enhance the well‐being of individuals with dementia, it is crucial to minimize the risk of deterioration in long‐term care needs. This study aimed to identify factors and construct predictive models for deterioration in long‐term care (LTC) levels in Japanese older adults with Mild Cognitive Impairment (MCI) and dementia.

**Method:**

This retrospective cohort study utilized the data from a memory clinic‐based cohort study (NCGG‐STORIES) and individual LTC insurance data provided by three municipalities. We included older adults aged ≥ 65 with MCI or dementia who had LTC levels ≤1 and resided in these municipalities at the initial visit. We used the certified LTC levels of the LTC insurance (seven care levels based on the severity of their physical and cognitive disability), and the outcome was the number of days from the initial visit to the new occurrence of LTC level ≥ 2, approximately equating to any care needs in basic activities of daily living (BADL). Demographic, lifestyle, functional, behavioral, and psychological factors assessed at the initial visit were included as potential predictors. The selection of predictors involved a backward stepwise Cox regression model, and the predictive model was formulated using a regression coefficient‐based scoring approach. Discrimination and calibration were assessed via Harrell’s C‐statistic and a calibration plot, respectively.

**Result:**

We analyzed 605 patients (mean age, 79.1 years; 57.7% women). During a mean follow‐up of 3.4 years, 329 older adults were newly certified LTC Level ≥ 2. Among possible sociodemographic and clinical variables, age, BADL, behavioral and psychological symptoms of dementia (BPSD), care burden, and history of fall were selected according to the analysis. The resulting predictive model exhibited good discrimination and calibration (Harrell’s C‐statistic [standard error] = 0.748 [0.013]).

**Conclusion:**

This study clarified some modifiable factors with deterioration in long‐term care needs, such as BPSD, care burden, and history of fall. Furthermore, the developed predictive model may have an effective discriminative ability for the new occurrence of LTC level ≥ 2 and holds potential for implementation in individuals with MCI and dementia referred to a memory clinic in Japan.